# Prediction of Discontinuation of Structured Exercise Programme in Chronic Fatigue Syndrome Patients

**DOI:** 10.3390/jcm9113436

**Published:** 2020-10-26

**Authors:** Sławomir Kujawski, Jo Cossington, Joanna Słomko, Helen Dawes, James W.L. Strong, Fernando Estevez-Lopez, Modra Murovska, Julia L. Newton, Lynette Hodges, Paweł Zalewski

**Affiliations:** 1 Department of Hygiene, Epidemiology, Ergonomy and Postgraduate Education, Ludwik Rydygier Collegium Medicum in Bydgoszcz Nicolaus Copernicus University in Torun, M. Sklodowskiej-Curie 9, 85-094 Bydgoszcz, Poland; jslomko@cm.umk.pl (J.S.); p.zalewski@cm.umk.pl (P.Z.); 2 Department of Sport, Health Sciences and Social Work, Centre for Movement Occupational and Rehabilitation Sciences, Oxford Brookes University, Oxford OX3 0BP, UK; jcossington@brookes.ac.uk (J.C.); hdawes@brookes.ac.uk (H.D.); 3 NIHR Oxford Health Biomedical Research Centre, Oxford OX3 7JX, UK; 4 Nuffield Department of Women’s & Reproductive Health, The Women Centre, University of Oxford, Oxford OX3 9DU, UK; jamie.strong@wrh.ox.ac.uk; 5 Department of Child and Adolescent Psychiatry/Psychology, Erasmus MC University Medical Center, PO Box 2040 Rotterdam, The Netherlands; fer@estevez-lopez.com; 6 Institute of Microbiology and Virology, Riga Stradiņš University, LV-1067 Riga, Latvia; modra@latnet.lv; 7 Population Health Science Institute, The Medical School, Newcastle University, Framlington Place, Newcastle-upon-Tyne NE2 4HH, UK; julia.newton@newcastle.ac.uk; 8 School of Sport, Exercise and Nutrition, Massey University, Palmerston North PN 621, New Zealand; L.D.Hodges@massey.ac.nz

**Keywords:** myalgic encephalomyelitis, chronic fatigue syndrome, autonomic nervous system, exercise, chronotropic intolerance, maximal heart rate, brain fog

## Abstract

Purpose: The purpose of this study was to assess differences in the physiological profiles of completers vs. non-completers following a structured exercise programme (SEP) and the ability to predict non-completers, which is currently unknown in this group. Methods: Sixty-nine patients met the Fukuda criteria. Patients completed baseline measures assessing fatigue, autonomic nervous system (ANS), cognitive, and cardiovascular function. Thirty-four patients completed a home-based SEP consisting of 10–40 min per day at between 30 and 80% actual HR max. Exercise intensity and time was increased gradually across the 16 weeks and baseline measures were repeated following the SEP. Results: Thirty-five patients discontinued, while 34 completed SEP. For every increase in sympathetic drive for blood pressure control as measured by the taskforce, completion of SEP decreased by a multiple of 0.1. For a 1 millisecond increase in reaction time for the simple reaction time (SRT), the probability for completion of SEP also decreases by a multiple of 0.01. For a one beat HRmax increase, there is a 4% increase in the odds of completing SEP. Conclusion: The more sympathetic drive in the control of blood vessels, the longer the reaction time on simple visual stimuli and the lower the HRmax during physical exercise, then the lower the chance of SEP completion in ME/CFS.

## 1. Introduction

Myalgic encephalomyelitis/chronic fatigue syndrome (ME/CFS) is a complex condition characterised by intense debilitating fatigue after physical activity. Individuals self-report symptoms including musculoskeletal pain, sleep disturbance, headaches, impairments in concentration and short-term memory [1]. Accumulating evidence suggests that the cardiovascular system may be compromised in individuals suffering with ME/CFS, along with reports of autonomic dysfunction [2], impaired heart rate, blood pressure regulation, and also impaired heart conduction [3]. Disturbance in response to physical exercise in ME/CFS is characterized by some patients as chronotropic intolerance, which is manifested by a lower maximal heart rate (HRmax) of less than 85% of age-predicted maximal exercise during a cardiopulmonary exercise test [4,5].

Previous studies have indicated the relationship between autonomic disturbance and cognitive dysfunction in ME/CFS [6]. Moreover, a decline in cognitive function has been noted after orthostatic stress, which is a provocative test for autonomic nervous system responsiveness [7]. Autonomic nervous system dysfunction is one of the most widely described parts of ME/CFS pathomechanisms [8]. Autonomic dysfunction could be related to a decrease in processing speed in ME/CFS [7]. Autonomic nervous system function was measured non-invasively using heart rate variability (HRV) as an indicator of cardiac autonomic innervation [7]. Processing speed could be measured by a simple reaction time test. Similar protocols based on this test have been applied in other patient groups [9]. The authors of meta-analysis describing pathological changes in autonomic cardiac innervation in ME/CFS suggested that the resting sympathetic hyperactivity, indicated by changes in HRV and blood pressure variability (BPV) might lead to a decrease in HRmax in ME/CFS patients [4]. Presumably, chronic sympathetic overactivity might lead to the downregulation of autonomic nervous system receptors and therefore lead to a decrease in HRmax. In recent years, treatment for ME/CFS has been largely focussed upon graded exercise therapy (GET) [10]. It was reported that 90% of examined ME/CFS patients suffer from post exertional malaise (PEM) [11]. Exacerbation of symptoms due to the physical or mental exercise in ME/CFS patients may potentially explain the statistically non-significant long-term effects of physical exercise programs on fatigue and disability compared to patients allocated to receiving standard medical care [12,13].

Exercise adherence is an important part of exercise prescription. PEM is a key symptom in individuals with ME/CFS [11]. It is important to consider this key symptom when prescribing an exercise programme for this population, as exercise may provoke these symptoms and as such decrease exercise adherence. It is important to note that in the PACE trial, exercise adherence was not directly measured, and only attendance was measured [14,15]. It was clear after 12 months of completing exercise prescription that there were minimal changes in fitness between the groups [15]. The lack of changes in fitness may suggest that there was a lack of adherence to the exercise programme; however, this was not directly reported [14,15]. The CONSORT statement on harms notes “it is important to report participants who are non-adherent or lost to follow up, because their actions may reflect their inability to tolerate the intervention” [16].

The purpose of this study is to address whether exercise adherence is a problem in this group of individuals and to gain an understanding of whether there is an underlying difference in physiology of those who complete the exercise trial compared with those who do not. We believe this is the first study of its kind and is of significant importance for this group.

## 2. Experimental Section

### 2.1. Participants

The study design was an exercise intervention study. The trial included two testing visits, once at baseline, and one post exercise intervention. The study was approved by the Ethics Committee, Ludwik Rydygier Memorial Collegium Medicum in Bydgoszcz, Nicolaus Copernicus University, Torun (KB 332/2013, date of approval: 25 June 2013); and written informed consent was obtained from all participants. Power calculation was based on the effect size of difference in HRpeak score in completers vs. non-completers using the pwr package in R environment. Assuming Cohen’s d = 0.68, the estimated power was 0.64.

#### Recruitment and Eligibility

**Figure 1:**
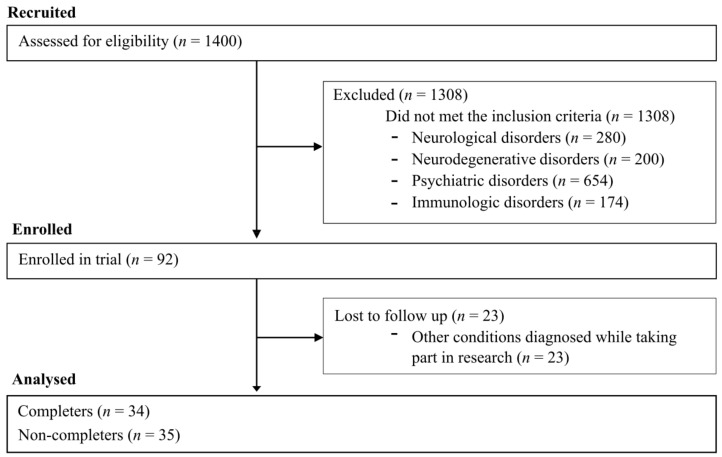
CONSORT-type flow diagram.

ME/CFS patients were included if they met the diagnostic criteria of the Fukuda case definition [17]. Initially, 1400 volunteers were assessed for eligibility in the trial, with 1308 being excluded. This left 69 individuals who met the trial inclusion criteria. However, only 53 patients undertook the structured exercise programme (SEP) protocol because 16 patients resigned from undertaking baseline cardiopulmonary exercise test (CPET). Thirty-four patients completed the protocol and n = 19 dropped out due to a reported severe post-exertional malaise (PEM) reaction to SEP (Figure 1).

### 2.2. Measures

#### 2.2.1. Fatigue Assessment Tools

The Chalder Fatigue Questionnaire (CFQ) was administered to assess fatigue severity. The questionnaire consists of 11 questions and is scored using a Likert scale of 0–3 (whereby 0 is the lowest, and 3 is the highest). Total or global score ranges from 0 to 33. The questionnaire is further split into two domains, physical fatigue (Q1–7) and psychological fatigue (Q8–11), which allows for the severity of physical and psychological fatigue to be assessed individually. Higher scores indicate higher symptom severity. The scale was validated in CFS patients [18].

The Fatigue Severity Score (FSS) contains 9 items examining the severity of factors related to fatigue during the past week. Scores range from 1 to 7, whereby 1 indicates strong disagreement and 7 strong agreement, with a higher score indicating a higher severity of fatigue [19].

The Fatigue Impact Scale (FIS) contains 40 items, scores range from 0 (no problem) to 4 (extreme problem) and is split into three domains of functioning; cognitive, physical and psychosocial. The higher the score, the more severe the symptoms. The total score ranges from 0 to 160 but can be divided into the three separate domains [20]. All questionnaires were administered at baseline and post-SEP intervention.

#### 2.2.2. Cognitive Function Measurement

Cognitive function was assessed using the computerized battery test described in more detail by Zalewski et al. (2020)—Test Sprawności Operacyjnej (TSO) (software version 4.6.0.44744, Speednet sp. z. o. o., more information available at http://www.biostat.com.pl/news/nowa_aplikacja_tso_stat_-181.php). TSO has been previously described in detail [21]. In brief, the simple reaction time (SRT) subtest involves the response to simple visual stimuli. Scores from the first attempt at the test after a short introduction were analysed.

#### 2.2.3. Autonomic Nervous System Measurement

Autonomic nervous system functioning was measured with Task Force Monitor—TFM (CNS Systems, Gratz, Austria) before more strenuous examinations to avoid confounding measured parameters. Signals from three-channel electrocardiogram (ECG) and continuous blood pressure monitoring (contBP with periodically cross-checked oscillometric blood pressure measurements) is analysed using the adaptive autoregressive model [22]. The TFM provides spectral analysis of blood pressure variability as indicators for sympathetic: low frequency (LF) and parasympathetic: high frequency (HF) regulation examination [23,24].

#### 2.2.4. Cardiopulmonary Exercise Testing

In the presence of a physician, the patients undertook a cardiopulmonary exercise test (CPET) using the Bruce protocol (Cardiovit CS-200 Ergo-Spiro, Schiller AG, Baar, Switzerland) at baseline [25,26]. A trained technician provided brief instruction and advised that the test would end voluntarily at the moment of subjective full exertion, or at any other time point, or at the command of the physician, using the guidelines for safe exercise testing by the American College of Sports Medicine [27]. As analysis of the VO2 plateau was omitted, a suffix “peak” is added to all variables measured during maximal workload during CPET. Heart rate (HR), oxygen consumption rate (VO2), carbon dioxide production rate (VCO2), minute ventilation (VE) and respiratory exchange ratio (RER (VCO2:VO2)) were measured to assess cardiopulmonary fitness at baseline (before SEP intervention) [25,26]. Chronotropic intolerance was defined if an actual HRpeak reached during CPET was lower than 85% of predicted maximal heart rate based on 220-age equation [5].

#### 2.2.5. Body Composition Analysis

To measure body composition a multi-frequency bioelectrical impedance analyser (Tanita MC-180MA Body Composition Analyzer, Tanita UK Ltd.) was used to assess weight (kg), fat mass (%), fat-free mass (%) and total body water (%). BMI (kg/m^2^) was automatically calculated by entering the subject’s height (cm), age (years) and gender.

### 2.3. Intervention

The SEP has been previously described in detail [21]. In brief, the programme consisted of a prescribed 16-week multimodal home exercise programme for 5 days a week, with time (10–40 min) and intensity (30–80% HRpeak) increasing gradually across the time period. Patients were equipped with heart rate monitors (Beurer PM 25) to help them in sustaining the recommended heart rate. HR intensity was individually prescribed based on the actual HRpeak achieved during the CPET. However, only 1 patient actually reached 80% of HRpeak during the last training sessions. Thirty-two patients reached 70%HRpeak, and 1 patient underwent last training sessions at 60%HRpeak. Participants were encouraged to undertake walking; however, cycling and swimming were allowed on a patients’ request. All subjects chose walking. Every week, telephone calls were made to resolve potential problems with compliance and to ensure patients were satisfied with the protocol. Patients underwent a minimum of 80 training sessions. The mean compliance rate was 80%. All of the analysed patients’ compliance rates were above 60%, which was a threshold value.

### 2.4. Statistical Analyses

Shapiro–Wilk test and histograms were used to test the assumption of normality. Mann–Whitney U or independent T-tests were used to examine between-group differences (completers vs. non-completers) depending on assumptions met. Benjamini–Hochberg adjusted *p* value was chosen to control for false discovery rate (FDR) in between-group comparisons. An online calculator available at https://www.sdmproject.com/utilities/?show=FDR was used to make a correction for the number of between-group comparisons made.

To predict SEP completion in examined patients, logistic regression model using glm function was applied in R. One cell with a lack of data in one of predictors was replaced with the mean value from the subgroup. In addition, 95% confidence intervals for log-likelihoods and odds ratios were calculated (confit function). DescTools package was used to calculate pseudo R2 for the model [28]. Dotwhisker plots were used to visualize odds ratios and confidence intervals [29]. Fisher’s exact test was applied to analyse the association between the presence of chronotropic intolerance with the non-completion of SEP.

## 3. Results

### 3.1. Comparison of Completers vs. Non-Completers before SEP

Thirty-four patients completed SEP while 35 did not. Data from n = 69 patients were analysed, except for CPET, where n = 19 in the non-completers group underwent this examination. There were 20 females in SEP completers subgroup and 21 in non-completers. A comparison of the body composition and age of completers vs. non-completers before SEP is presented in Table 1. No significant difference was observed in age, BMI, free fat mass (FFM) or in fat percentage.

A comparison of the fatigue of completers vs. non-completers before SEP is presented in Table 2. No significant difference was observed in fatigue before SEP in completers vs. non-completers.

The comparison of autonomic nervous system functioning before SEP is presented in Table 3. Non-completers had significantly higher LFnu-sBP (46.99 (13.2)% vs. 37.14 (12.1)%, Z = 2.81, *p* = 0.005, r = 0.34) and reaction time in the SRT.1 (647.91 (174.1) ms vs. 547.09 (162.6) ms, Z = 3.11, *p* = 0.002, r = 0.38). However, the results are not significant after FDR correction.

Nineteen non-completers underwent CPET before SEP. No significant differences between groups were found in CPET parameters during AT (Table 4).

Chronotropic intolerance was noted in four patients in the completers group (11.76%) compared to seven patients (20%) in the non-completers group (*p* = 0.04). Non-completers had significantly lower RERpeak (1.08 (0.1) % vs. 1.15 (0.1), Z = −2.12 *p* = 0.03, r = −0.29. However, this comparison was not significant after FDR correction (Table 5). Nine out of 19 patients in the non-completers group (47.37%) did not reach RERpeak ≥ 1.1, while 6 patients from 34 (17.65%) in the completers group did not reach this threshold.

### 3.2. Predictors of SEP Completion

**Figure 2:**
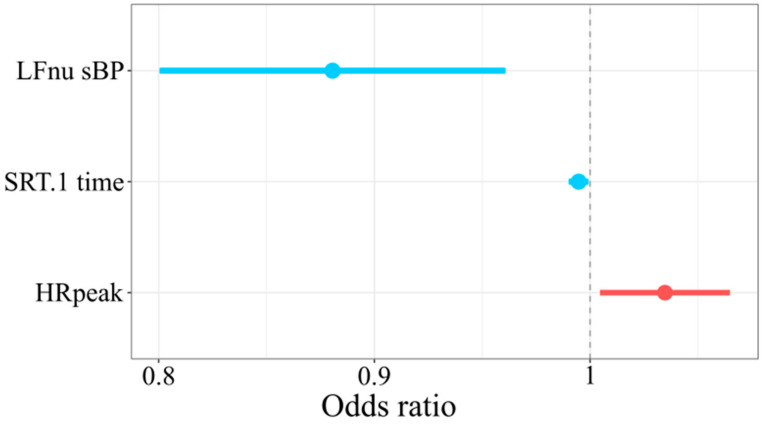
Odds ratio for SEP completion. Horizontal axis refers to odds ratio. Dots denote odds ratio, while horizontal lines nearby dots denote −95 and 95% of odds ratio confidence intervals.

Table A1 presents the results of the logistic regression model predicting SEP completion. For normalized low frequency systolic blood pressure unit increase, the probability of the completion of SEP decreases by a multiple of 0.11. For the increase in the reaction time in the first attempt to SRT, the probability of the completion of SEP decreases by a multiple of 0.01. For a one beat per minute more of heart rate during maximal physical exercise, an increase about 4% in the odds of completing SEP is expected (AIC = 54.07, BIC = 61.96, Tjur R2 = 0.38) (Figure 2).

## 4. Discussion

This study demonstrates that the more the sympathetic drive in the control of blood vessels as measured by TFM (low frequency systolic blood pressure), the longer the reaction time on simple visual stimuli measured by simple reaction time test reflecting slower cognitive functioning and the lower maximal heart rate during physical exercise, then the less chance there is of SEP completion. Moreover, chronotropic intolerance occurred more frequently in non-completers than in completers. However, what should be underlined is that we have noted a considerable withdrawal rate (35 from 69 patients). Sixteen resigned from CPET during baseline, due to the fear of exacerbating symptoms of post-exertional malaise. Of those remaining, 19 undertook the baseline CPET but dropped out of the SEP due to adverse effects, leaving 34 who completed the SEP.

### 4.1. Withdrawal Rate

Whilst withdrawals are completely normal during supervised exercise programmes, when comparing this rate in the current study (23% pre CPET testing, 36% after CPET testing) to those in previously reported studies within ME/CFS, it is interesting to note that this is substantially larger than that evidenced in the GET trial (6%) [10], and the GETSET study (12 participants, 6%) of 211 participants [30]. In the current study, the mean score of CFQ before SEP 26.12, which is comparable to both the GET group in the PACE trial (28.2 points) [31] and for the GET group in the GETSET trial (26.3) [10]. In addition, we found no significant differences between our groups of completers and non-completers. Therefore, higher dropout rate in the above study is not explained by greater fatigue before physical exercise programmes. It could be speculated that the above sample suffered from more frequent and/or intense PEM; however, it was not measured by any scale. Therefore, no strong conclusions could be drawn based on dropout rate noted in the above study.

### 4.2. Differences between SEP Completers and Non-Completers

Respiratory exchange ratio is one of the best indicators of effort during physical exercise. Maximal effort is indicated by RER ≥ 1.1 [27]. In the above study, SEP non-completers were characterized by lower RER max than completers. In contrast with the above findings, all 22 examined ME/CFS patients’ RER max scores were high (≥1.1) [32]. Moreover, RER max was reproduced 24 h during the following CPET test [32]. Moreover, Oosterwijck et al. reported that the mean RER max as 1.25 (0.98) in 22 women with ME/CFS [33], which was higher than RER max in a control group consisting of sedentary subjects (mean 0.92 (0.11). In another study, patients were divided into three subgroups according to symptom severity (severe, moderate and mild) and underwent 2-day CPET protocol [34]. RER max was 1.08 (0.09) in the severe group, 1.09 (0.09) in the moderate and 1.13 (0.11) in the mild subgroups in the first CPET. In our study, non-completers also reached a mean of 1.08 RER max, similarly to the most severe group in van Campen et al. The Authors [34] suggests that for patients in a severe group, skeletal muscle exhaustion might occur faster than limitations of central hemodynamic and ventilatory origins. In line with that explanation, we also speculate that non-completers in the current study perceived more intense adverse effects during physical exercise and therefore ended more quickly.

What is interesting is that we have noted no statistically significant results in VO2max between completers and non-completers. However, the observed differences suggest that clinically significant better VO2max in completers vs. non-completers, according to the definition of minimum clinically important difference between CFS and controls, is of 1.1 mL/kg/min [35]. Mitochondrial dysfunction is suggested as one of the main factors in ME/CFS pathomechanism [36]. In EACPR/AHA, the Scientific Statement suggests that the measurement of the ratio of maximal cardiac output to VO2max in patients with suspected mitochondrial myopathy is useful in diagnosis [37]. Therefore, we suggest that further studies on ME/CFS should focus on including the wide assessment of multiple systems functioning in response to physical exercise.

### 4.3. Predictors of SEP Completion

This study is the first to analyse the predictors of completion of a multimodal supervised exercise program in ME/CFS patients. It is evident in the examined sample that the greater sympathetic drive in control of blood vessels, the longer reaction time on simple visual stimuli, and lower maximal heart rate levels found during physical exercise, are linked to lower chance of SEP completion. Recent meta-analysis has described disturbance in the autonomic control over heart rate in ME/CFS; however, it is important to be able to distinguish whether this involves all individuals with diagnosed ME/CFS or whether this is indeed a subset of those individuals. It is becoming increasingly evident that ME/CFS participants are characterized by lower maximal heart rate during physical exercise sessions [4]. This phenomenon is described as chronotropic intolerance [5]. In our current study, it can be seen that completers demonstrated a peak HR of 93% of age predicted max, whilst the non-completers demonstrated a peak HR of 86%, therefore demonstrating that the non-completers showed evidence of chronotropic intolerance. It has also been demonstrated that there is aa disturbance in the autonomic control of blood vessels, which may be related to a worsening response to physical exercise in ME/CFS patients. During physical exercise, blood is redistributed to supply working muscles. However, a recent study found endothelial dysfunction in ME/CFS [38]. Taking into account methodological problems of non-invasive autonomic nervous system function monitoring, low frequency systolic blood pressure might be a more precise indicator of sympathetic activity than low frequency of heart rate [39,40,41].

Brain fog/cognitive function disturbance is one of the most prominent symptoms in patients with ME/CFS, occurring in 85–95% [42,43]. In the current study, those who withdrew from the supervised exercise displayed worse cognitive function than those who completed the exercise, which indicates that cognitive dysfunction might coexist with a poor response to physical exercise in ME/CFS subjects. Coexistence of autonomic disturbance and cognitive dysfunction in ME/CFS was noted in previous studies [7,8]. On the other hand, it might be speculated that nervous system disturbances might play a role in PEM pathomechanism. Therefore, we suggest including both cognitive and autonomic along with PEM assessment into ME/CFS patients routine diagnosis.

### 4.4. Study Limitations

We have noted a considerable withdrawal rate (35 from 69 patients). Sixteen patients resigned from CPET during baseline and therefore we were unable to incorporate this subgroup in all comparisons. Moreover, in the above study, PEM was not measured. Due to the relatively small sample size, results on differences SEP completers and non-completers should be replicated in further studies. A potential future research study should incorporate a questionnaire assessing PEM in ME/CFS patients undergoing aerobic exercise program. Moreover, the effects of personalizing the intensity of an exercise program based on CPET result and PEM should be examined.

## 5. Conclusions

In conclusion, this study is the first to demonstrate possible physiological reasons for participant withdrawal from studies which use the NICE endorsed physical exercise programme. However, due to the small sample size in this study, this study should be replicated.

This study demonstrated that structured exercise programmes may not be suitable for all individuals with ME/CFS. It is apparent that there may be a subgroup of individuals who have chronotropic intolerance, as demonstrated through a single CPET test. Further studies should focus on whether these individuals should avoid supervised exercise programmes as well as elucidating other important markers such as CPET testing results, autonomic and cognitive function, amongst others which may further help in personalizing therapy for ME/CFS subgroups.

## Figures and Tables

**Table 1:** Comparison of completers vs. non-completers in body composition before structured exercise programme (SEP).

Variable	Mean (SD) Non-Completers (*n* = 35)	Mean (SD) Completers (*n* = 34)	*p* Value	FDR Adjusted *p* Value
Age (years)	39.6 (9)	37.06 (7.9)	0.25	0.57
BMI (kg/m^2^)	24.02 (3.5)	24.52 (3.2)	0.54	0.86
FFM (kg)	53.76 (11)	54.45 (9.7)	0.78	0.99
Fat (%)	24.2 (7)	25.04 (6.6)	0.61	0.92

BMI—body mass index, FFM—free-fat mass.

**Table 2:** Comparison of completers vs. non-completers in fatigue before SEP.

Variable	Mean (SD) Non-Completers (*n* = 35)	Mean (SD) Completers (*n* = 34)	*p* Value	FDR Adjusted *p* Value
CFQ (points)	24.54 (3.9)	26.12 (3.5)	0.08	0.38
FSS (points)	48.74 (8.7)	48.91 (8.9)	0.94	0.99
FIS (points)	90.91 (27.7)	93.59 (24.9)	0.67	0.96

CFS—Chronic Fatigue Questionnaire, FSS—Fatigue Severity Scale, FIS—Fatigue Impact Scale.

**Table 3:** Comparison of completers vs. non-completers in autonomic nervous system and cognitive functioning before SEP.

Variable	Mean (SD) Non-Completers (*n* = 35)	Mean (SD) Completers (*n* = 34)	*p* Value	FDR Adjusted *p* Value
LFnu-RRI (%)	51.62 (19)	56.81 (14.9)	0.21	0.57
HFnu-RRI (%)	48.38 (19)	43.19 (14.9)	0.21	0.57
LF/HF-RRI	1.55 (1.3)	1.91 (2)	0.40	0.69
LF/HF	1.42 (0.8)	1.53 (1.3)	0.82	0.99
LFnu-dBP (%)	54.24 (14.9)	50.06 (14.4)	0.24	0.57
HFnu-dBP (%)	14.23 (11.4)	13.73 (9.4)	0.78	0.99
LFnu-sBP (%)	46.99 (13.2)	37.14 (12.1)	0.005	0.08
HFnu-sBP (%)	16.61 (10)	16.46 (11.6)	0.95	0.99
SRT.1 mean reaction time (ms)	647.91 (174.1)	547.09 (162.6)	0.002	0.07

LFnu-RRI (%)—low-frequency normalized units of R-R interval, HFnu-RRI (%)—high-frequency normalized units of R-R interval, LF/HF-RRI—ratio of LF to HF of R-R interval, LF/HF—ratio of LF-dBP to HF-RRI, LFnu-dBP (%)—low-frequency normalized units of diastolic blood pressure, HFnu-dBP (%)—high-frequency normalized units of diastolic blood pressure, LFnu-sBP (%)—low-frequency normalized units of systolic blood pressure, HFnu-sBP (%)—high-frequency normalized units of systolic blood pressure, SRT.1 mean reaction time (ms)—mean reaction time in the first attempt to Simple Reaction Time test.

**Table 4:** Comparison of completers vs. non-completers before SEP in cardiopulmonary exercise test (CPET) parameters during AT.

Variable	Mean (SD) Non-Completers (*n* = 19)	Mean (SD) Completers (*n* = 34)	*p* Value	FDR Adjusted *p* Value
VO2 AT [mL/kg/min]	21.89 (5.9)	21.86 (4.8)	0.99	0.99
VCO2 AT [mL/kg/min]	21.21 (6.7)	21.36 (4.9)	0.93	0.99
RER AT	0.96 (0.1)	0.98 (0)	0.95	0.99
VE AT [mL/kg/min]	602.84 (190.8)	579.86 (128.8)	0.24	0.57
HR AT [bpm]	136.00 (22.5)	144.21 (16.4)	0.14	0.57
Load AT [W]	102.28 (22.3)	99.26 (44.4)	0.31	0.60
sBP AT [mmHg]	161.72 (51.5)	167.18 (23.8)	0.99	0.99
dBP AT [mmHg]	81.39 (24.1)	88.59 (14.9)	0.55	0.86

VO2/kg AT (ml/min/kg)—oxygen consumption during anaerobic threshold, VCO2/kg AT (ml/min/kg)—carbon dioxide production during anaerobic threshold, RER AT—ratio of VCO2 to VO2 during anaerobic threshold, VE/kg AT [ml/kg/min]—minute ventilation during anaerobic threshold, HR AT [bpm]—heart rate during anaerobic threshold, Load AT [W]—load during anaerobic threshold, sBP AT [mmHg]—systolic blood pressure during anaerobic threshold, dBP AT [mmHg]—diastolic blood pressure during anaerobic threshold.

**Table 5:** Comparison of completers vs. non-completers before SEP in CPET parameters during maximal exercise.

Variable	Mean (SD) Non-Completers (*n* = 19)	Mean (SD) Completers (*n* = 34)	*p* Value	FDR Adjusted *p* Value
VO2peak [mL/min/kg]	26.33 (7.4)	29.49 (6.3)	0.07	0.38
VCO2peak [mL/min/kg]	29.41 (9.8)	34.04 (7.7)	0.06	0.38
RERpeak	1.08 (0.1)	1.15 (0.1)	0.03	0.33
VEpeak [mL/kg/min]	885.09 (318.8)	968.35 (239.6)	0.29	0.60
HRpeak [bpm]	166.39 (25)	182.35 (21.9)	0.06	0.38
Load peak [W]	119.84 (47.6)	132.94 (34.6)	0.26	0.57
sBPpeak [mmHg]	191.58 (35.0)	180.94 (26.7)	0.22	0.57
dBPpeak [mmHg]	90.53 (13.5)	90.65 (11.1)	0.97	0.99

VO2/kg peak (ml/min/kg)—oxygen consumption during maximal intensity exercise, VCO2/kg peak (ml/min/kg)—carbon dioxide production during maximal intensity exercise, RER peak—ratio of VCO2 to VO2 during maximal intensity exercise, VE/kg peak [ml/kg/min]—minute ventilation during maximal intensity exercise, HR peak [bpm]—heart rate during maximal intensity exercise, Load peak [W]—load during maximal intensity exercise, sBP peak [mmHg]—systolic blood pressure during maximal intensity exercise, dBP peak [mmHg]—diastolic blood pressure during maximal intensity exercise.

## Data Availability

Individual data is available from the corresponding author S.K. on request.

## Appendix A

**Table A1:** Logistic regression coefficients predicting SEP completion.

Variable	Estimate	−95% CI	95% CI	z Value	Pr(>|z|)
(Intercept)	3.15	−3.07	9.38	0.99	0.32
LFnu sBP	−0.12	−0.20	−0.04	−2.92	0.003
SRT.1 reaction time	−0.01	−0.01	−0.0008	−2.29	0.02
HRmax baseline CPET	0.03	0.005	0.06	2.26	0.02
